# Sex-differential non-specific effects of adjuvanted and non-adjuvanted rabies vaccines versus placebo on all-cause mortality in dogs (NERVE-Dog study): a study protocol for a randomized controlled trial with a nested case–control study

**DOI:** 10.1186/s12917-022-03455-6

**Published:** 2022-10-01

**Authors:** Darryn L. Knobel, Anne Conan, Felix N. Toka, Sintayehu M. Arega, Charles Byaruhanga, Eric Ogola, Erick M. O. Muok, Jan E. Crafford, Andrew L. Leisewitz, Melvyn Quan, Mary Anna Thrall

**Affiliations:** 1 Department of Biomedical Sciences, Ross University School of Veterinary Medicine, Basseterre, St Kitts and Nevis; 2 Department of Veterinary Tropical Diseases, Faculty of Veterinary Science, University of Pretoria, Onderstepoort, South Africa; 3 Center for Applied One Health Research and Policy Advice, City University of Hong Kong, Kowloon, Hong Kong, Special Administrative Region of China; 4 Department of Public and Community Health, Jaramogi Oginga Odinga University of Science and Technology, Bondo, Kenya; 5 Centre for Global Health Research, Kenya Medical Research Institute, Kisumu, Kenya; 6 Department of Companion Animal Clinical Studies, Faculty of Veterinary Science, University of Pretoria, Onderstepoort, South Africa; 7 Present Address: Department of Clinical Sciences, College of Veterinary Medicine, Auburn University, Auburn, USA

**Keywords:** Non-specific effects, Vaccine, Sex, Mortality, Rabies, Dogs, Cytokines, Haemoparasites, Viruses, Helminths

## Abstract

**Background:**

It has been proposed that childhood vaccines in high-mortality populations may have substantial impacts on mortality rates that are not explained by the prevention of targeted diseases, nor conversely by typical expected adverse reactions to the vaccines, and that these non-specific effects (NSEs) are generally more pronounced in females. The existence of these effects, and any implications for the development of vaccines and the design of vaccination programs to enhance safety, remain controversial. One area of controversy is the reported association of non-live vaccines with increased female mortality. In a previous randomized controlled trial (RCT), we observed that non-live alum-adjuvanted animal rabies vaccine (ARV) was associated with increased female but not male mortality in young, free-roaming dogs. Conversely, non-live non-adjuvanted human rabies vaccine (NRV) has been associated with beneficial non-specific effects in children. Alum adjuvant has been shown to suppress Th1 responses to pathogens, leading us to hypothesize that alum-adjuvanted rabies vaccine in young dogs has a detrimental effect on female survival by modulating the immune response to infectious and/or parasitic diseases. In this paper, we present the protocol of a 3-arm RCT comparing the effect of alum-adjuvanted rabies vaccine, non-adjuvanted rabies vaccine and placebo on all-cause mortality in an owned, free-roaming dog population, with causal mediation analysis of the RCT and a nested case–control study to test this hypothesis.

**Methods:**

Randomised controlled trial with a nested case–control study.

**Discussion:**

We expect that, among the placebo group, males will have higher mortality caused by higher pathogen loads and more severe disease, as determined by haematological parameters and inflammatory biomarkers. Among females, we expect that there will be no difference in mortality between the NRV and placebo groups, but that the ARV group will have higher mortality, again mediated by higher pathogen loads and more severe disease. We anticipate that these changes are preceded by shifts in key serum cytokine concentrations towards an anti-inflammatory immune response in females. If confirmed, these results will provide a rational basis for mitigation of detrimental NSEs of non-live vaccines in high-mortality populations.

## Background

Vaccines work by stimulating the body to produce a high-quality, rapid and specific immune response in recipients upon exposure to infection by a particular pathogen or closely related group of pathogens. Recently, it has been proposed that vaccines may have additional, unanticipated effects on the immune system that manifest as a general increase or decrease in morbidity or mortality in host populations, which cannot be explained by the specific protective effect induced by the vaccine against the target pathogen(s), nor conversely by typical expected adverse reactions to the vaccine [1–3]. These effects of vaccines have been termed “non-specific effects” (NSEs). There is now considerable evidence in the public health literature that vaccination alters susceptibility to unrelated (heterologous) infections, and that these NSEs differ by vaccine type (live vs. non-live) and by sex (male vs. female) [4]. Commissioned by the World Health Organisation (WHO), a systematic review of the association of Bacillus Calmette-Guérin (BCG), measles-containing vaccines (MCV) and diphtheria-tetanus-pertussis (DTP) vaccine with all-cause mortality in children under 5 years of age concluded that the evidence indicated that BCG and MCV (both live vaccines) reduce overall mortality by more than would be expected through their specific protective effects, but that receipt of non-live DTP may be associated with an increase in all-cause mortality [5]. The study authors recommended further randomized controlled trials, and that these trials should be sufficiently powered to examine possible differential effects between boys and girls [5]. The findings of the systematic review are supported by studies showing that other live vaccines such as oral polio vaccine and smallpox vaccine reduce all-cause mortality [6–9] while other non-live vaccines including the H1N1 influenza vaccine [7], inactivated polio vaccine [10], a hepatitis B vaccine [11], a pentavalent (DTP, hepatitis B and *Haemophilus influenzae b*) vaccine [12, 13], and the RTS/AS01 malaria vaccine [14, 15] have all been associated with higher mortality in girls than in boys [4]. There are now ten studies with prospective follow-up comparing DTP-vaccinated with DTP-unvaccinated children, and in a combined analysis vaccinated children had 2.07 (95% CI 1.60–2.67) times higher average all-cause mortality than unvaccinated children, with vaccinated girls having 1.47 (1.18–1.84) higher mortality than boys [4, 16]. In contrast, findings for BCG vaccine show that beneficial effects are more pronounced in boys [17].

To date, few studies have explored the mechanistic link between vaccination and non-specific outcomes [18]. In an effort to identify the biological mechanism of the beneficial effect of BCG vaccine on the incidence of heterologous infectious diseases in Ugandan neonates, Prentice et al. [17] showed that vaccination was associated with epigenetic changes of cytokine promoters in peripheral blood mononuclear cells but could not demonstrate that these changes mediated the effect of BCG on the outcome, nor whether these epigenetic changes altered cytokine production. Other studies report increased proinflammatory cytokine production to heterologous stimuli in neonates after BCG vaccination, although the significant stimuli and cytokines varied [19, 20]. Alternative possible mechanistic explanations for the beneficial effect of BCG include induction of emergency granulopoiesis [21] and enhanced heterologous T-cell responses [22]. By contrast, we can identify only one study that examined a biological mechanism for the detrimental effect of non-live vaccines. In that study, Noho-Konteh et al. [23] found that compared to controls, DTP-vaccinated females in particular had lower proinflammatory responses to TLR4 stimulation and suppressed T-cell reactivity. A typical feature of non-live vaccines is the addition of an adjuvant to enhance the immunogenicity of the vaccine antigen [24], with aluminium-containing adjuvants being the most widely used [25]. Alum adjuvant has been shown to suppress antigen-specific Th1 responses through enhanced production of anti-inflammatory cytokines [25–29]. We hypothesize that this immunosuppressive effect of alum adjuvant may extend to suppression of proinflammatory responses to concurrent or subsequent unrelated infections. This hypothesis could also explain the sex-differential nature of the detrimental effects of non-live vaccines, in that males already have suppressed proinflammatory responses compared to females [30, 31], and thus inhibition of proinflammatory responses is more marked in females.

There are considerable methodological and ethical challenges to the study of NSEs of vaccines that are in routine use in populations with high infant and child mortality rates [32–35]. Consequently, much of the evidence for vaccine NSEs is derived from observational studies with a high risk of bias, and little from high-quality randomized controlled studies testing pre-specified hypotheses. We propose to study NSEs of non-live vaccines in young, owned free-roaming domestic dogs, using rabies vaccines as a model. Although primary vaccination of dogs against rabies is recommended no earlier than 12–14 weeks of age by vaccine manufacturers, current guidance from the WHO [36] and the World Organisation for Animal Health (OIE) [37] allow for adaptation of vaccination schedules to include vaccination of dogs younger than 12 weeks. Vaccination of dogs at around 6 weeks of age is therefore common practice in the context of mass rabies vaccination campaigns, and this will be the target age group for the proposed study (6 to 13 weeks of age). Because of their relatively high burden of infectious and parasitic diseases and low routine vaccination coverage, young, owned free-roaming dogs in low-resource communities provide an opportunity to study the NSEs of different vaccine types and further elucidate the mechanisms of action of NSEs. Populations with high infectious and parasitic disease burdens also stand to gain most benefit if the NSEs of vaccines could be understood and harnessed to enhance protection against these diseases.

Rabies vaccines are inactivated vaccines that are typically but not always combined with an alum adjuvant. Notably, Gessner et al. [38, 39] reported that a non-adjuvanted rabies vaccine (NRV) was associated with a lower risk of heterologous central nervous system infections (meningitis and cerebral malaria) in children. Older studies with non-adjuvanted live-attenuated or partially inactivated rabies vaccines were also suggestive of protective NSEs [40, 41]. In an observational study, we found that owner-reported vaccination of dogs with an alum-adjuvanted rabies vaccine (ARV) was also associated with a beneficial NSE, lowering the risk of death from any cause [42]; however, it has been shown that effect estimates based on recording vaccination status at successive visits, in which information on vaccinations that occurred between visits is updated at the time of the second visit (so-called “retrospective updating”, as we did in the observational study), can lead to considerable bias in vaccine studies, biasing observed mortality rate ratios towards zero (a beneficial effect) [33]. Recognizing that our analysis of the observational study data was at risk of bias, we conducted an owner-blinded randomized controlled animal trial (RCT) of the effect of ARV on all-cause mortality in young dogs in the same population [43]. In contrast to our previous observational study, the RCT showed that ARV substantially increased all-cause mortality in female but not male dogs, thus mirroring the effect seen for DTP and other non-live vaccines in children in similar populations [4, 16].

To resolve the discrepancy between the disparate findings across previous studies of NSEs of rabies vaccines, we propose to conduct a 3-arm RCT comparing the effect of ARV, NRV and placebo on all-cause mortality in an owned, free-roaming dog population in a different site. The objectives of the study are to determine if survival time for all causes of death differs between treatment groups, and if that effect is modified by sex. The study will also determine to what extent the effects of treatment group and sex on survival time are mediated by the nature of the immune response elicited, as measured by plasma cytokine concentrations 24 h after treatment. A nested case–control (NCC) study will aim to determine if effects on survival time are mediated through changes in susceptibility to common infectious and parasitic diseases, as determined by quantitative polymerase chain reaction (qPCR) for aetiologies of common viral [*Canine protoparvovirus* (CPV) and *Canine morbillvirus* (canine distemper virus, CDV)], haemoparasitic (*Babesia* spp., *Ehrlichia canis* and *Anaplasma platys*) and helminth (*Ancylostoma* sp. and *Toxocara canis*) infections in samples collected at specified time points, or by changes in haematological parameters or plasma c-reactive protein (CRP) concentrations at these same time points. Through this study, we hope to understand the mechanism of action of any non-specific effects of rabies vaccines, so that potentially beneficial effects might be harnessed and any detrimental effects mitigated.

## Methods/Design

### Study objectives

#### Primary objective

1. To determine if the effect of (i) ARV compared to placebo and (ii) NRV compared to placebo on survival time in 6-week-old puppies is modified by sex.

The primary null hypotheses are:

1.1. There is no modification by sex of the effect of ARV compared to placebo on survival time
1.2. There is no modification by sex of the effect of NRV compared to placebo on survival time

#### Secondary objectives

1. To determine the extent to which the effect of (i) ARV compared to placebo and (ii) NRV compared to placebo on survival time in 6-week-old puppies is mediated by the Th1/Th2 plasma cytokine balance score 24 h after injection, in male dogs and female dogs separately.
2. To determine the extent to which the effect of (i) ARV compared to placebo and (ii) NRV compared to placebo on survival time in 6-week-old puppies is mediated by changes in individual cytokine plasma concentrations 24 h after injection, in male dogs and female dogs separately.
3. To determine the extent to which the effect of (i) ARV compared to placebo and (ii) NRV compared to placebo on survival time in 6-week-old puppies is mediated by the following parameters, in male dogs and female dogs separately:
  a body weight
  b abdominal girth
  c modified Volhard’s puppy aptitude test score
  d haemoparasite load in peripheral blood (*Babesia, Ehrlichia, Anaplasma*)*
  e viral load in rectal swabs (CDV, CPV)*
  f parasite load in rectal swabs (*Ancylostoma, Toxocara, Giardia, Coccidia*)*
  g haematological parameters (including haematocrit, erythrocyte concentration, haemoglobin concentration, erythrocyte mean cell volume, reticulocyte concentration, and reticulocyte haemoglobin content)
  h plasma c-reactive protein concentration* (*NCC only)
4. To determine if cause-specific mortality rates differ between treatment groups and by sex.
5. To determine if sex affects survival time from birth to six weeks of age.

### Study design

The NERVE-Dog animal trial is designed as a randomized, placebo-controlled, owner-blind, single centre comparative trial with three parallel groups with a 1:1:1 allocation ratio, stratified by litter, restricted to puppies born to females vaccinated against rabies within the preceding 18 months. A nested case–control study will allow evaluation of additional potential mediators of the effect of treatment group on the outcome.

To identify eligible puppies at 6 weeks of age, adult female dogs (and their future offspring) resident in the study area will be enrolled and followed until birth. From this birth cohort, puppies eligible for the NERVE-Dog trial will be identified, followed and randomized at 42 days of age.

### Description of the study population

**Fig. 1:**
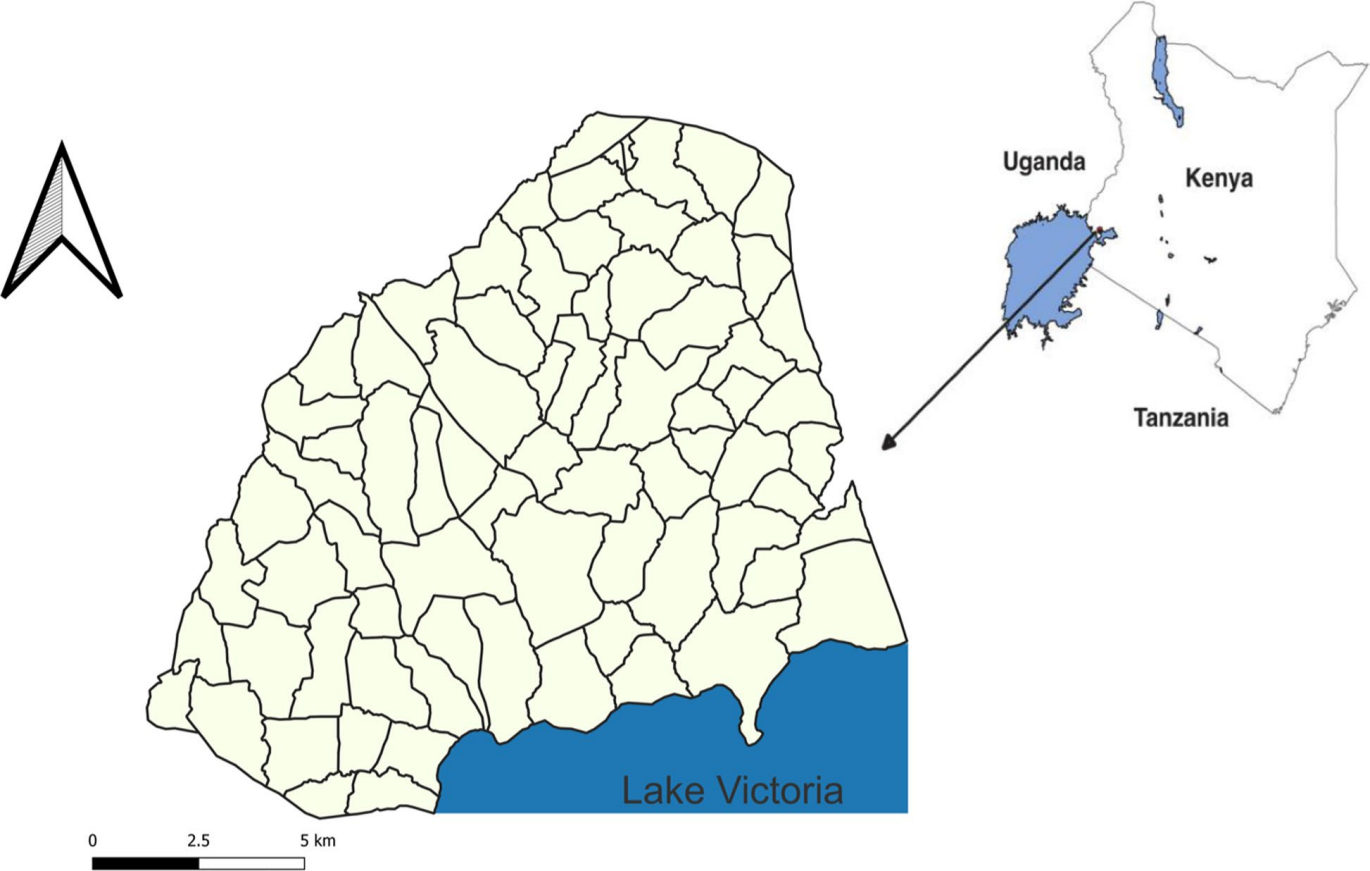
A map of Kenya showing the location of the study site. The image is our own

The site of the study (Fig. 1) will be 33 villages in Asembo Division, Rarieda Sub-county, Siaya County, Kenya. Siaya is one of the five counties earmarked for piloting rabies elimination in Kenya by the year 2030 (Strategic Plan for the Elimination of Human Rabies in Kenya, 2014 – 2030; retrieved from https://caninerabiesblueprint.org/Strategic-Plan-for-the-Elimination). It comprises Bondo, Alego-Usonga, Ugenya, Gem, Ugunja and Rarieda Sub-counties. Rarieda Sub-county is included in a human Health and Demographic Surveillance System (HDSS) [44].

Study animals will be privately-owned puppies, recruited from litters born to owned adult female dogs resident in the study site. Puppies will be housed and managed by their owners for the duration of the study and will remain with their owners upon exit from the study. The total human population in Siaya County is estimated at 993,183 individuals [45] comprising about 220,000 households. The primary language spoken is Dholuo. Around 38% of households in Asembo own dogs, with a dog:human ratio of 1:7 and an average of 1.8 adult dogs per dog-owning household [46]. The majority of dogs have the phenotypic appearance of the Africanis landrace.

### Inclusion and exclusion criteria

Identification of eligible puppies requires accurate information about the puppies’ dates of birth and the rabies vaccination status of the puppies’ mothers at the time of birth. The accuracy of this information will be substantially enhanced by capturing it at the time of birth, rather than at the time of randomization (6 weeks of age), due to unreliable owner recall. Thus, enrolment of prospective participating puppies will begin with the enrolment of prospective mothers (adult female dogs) into the study. Adult female dogs will be followed up until they give birth. From this birth cohort, puppies eligible for the NERVE-Dog trial will be identified, followed up and randomized at 6 weeks (42 days) of age.

Owner recruitment will take place over a period of 3 months. Identification of owners of eligible dogs will be done through house-to-house visits, discussions with key informants, and ‘snowball sampling’ where dog owners will be asked to identify other dog owners in their village.

Owners must provide written, informed consent before any study procedures occur, in adult females (observational only) or puppies (observational only until 6 weeks of age; thereafter interventional). The study consent form is written to be readable at a primary education level (Flesch-Kincaid grade level < 7). The consent form has been translated into the Dholuo language. Owners will be asked if they would prefer a copy of the informed consent form in English or Dholuo to read (for those owners with a level of education higher than primary), or to have it read to them. If the latter, the consent document will be read to the participant by a member of the study team, in the presence of a witness.

#### Inclusion criteria for enrolment of adult female dogs

To be eligible for inclusion, female dogs must meet all of the following criteria at the time of enrolment:

1. Resident within the study area;
2. Owner-reported age ≥ 12 months.

#### Exclusion criteria for enrolment of adult female dogs

Adult female dogs will be excluded from the study if they meet any of the following criteria:

1. The owner applies fertility control methods to the dog, including surgical sterilization, hormonal contraception, or strict confinement during oestrus (heat);
2. The owner is unable to produce a valid certificate of vaccination within the last 12 months against rabies, and the owner is unwilling to have the dog vaccinated against rabies for enrolment into the study;
3. The owner does not provide written informed consent for participation in the study.

For owners of eligible adult female dogs who provide informed consent to participate in the study, project personnel will vaccinate all dogs in the household against rabies, as necessary. Project personnel will also monitor the vaccination status of all dogs in participating households and revaccinate as necessary (every 12 months). In this way, all adult females will have been vaccinated against rabies within 12 months before birth. All information on vaccinations done by study personnel will be provided to the Subcounty Veterinary Officer.

#### Inclusion criteria for puppies in NERVE-Dog trial:

To be eligible for inclusion in the NERVE-Dog trial, puppies must meet all of the following criteria at randomization:

1. Born to female dogs resident in the study area;
2. Female dog vaccinated against rabies within 12 months before the birth;
3. Date of birth recorded (within ± 3 days);
4. Followed up since birth;
5. 39–45 days of age on the day of the intervention.

Change of ownership between birth and 6 weeks of age will not exclude puppies from the NERVE-Dog trial if they meet the above criteria and both of the following criteria:

1. The new owner is resident within the study area;
2. The new owner provides written, informed consent for participation in the study.

### Randomisation method

#### Sequence generation

Randomisation will be stratified by litter at 42 days of age. Litter size (*n*) will be ascertained from the owner one to two days before randomisation. Before the allocation visit on study day 0, *n* number of microchips will be selected and the allocation code assigned to each microchip number by computer-generated random numbers (using the *crPar* function for 3 treatment groups in the package ‘randomizeR’ [47] in R statistical software [48]).

#### Concealment mechanism

The microchip numbers and corresponding allocation group letter will be written in a table on the Allocation Visit form for Study Day 0. The microchips will be placed in an opaque bag. Within two hours before the allocation visit, the number of interventions for the litter will be prepared in identical syringes (1 mL in each syringe). Opaque tape will be placed around the syringe to conceal the contents, and the allocation group letter will be written on the tape. Prepared syringes will be placed in a cool box with an ice pack. Syringes will be wrapped in paper towel to prevent direct contact with the ice pack. At the time of allocation, a member of the study team will select a puppy for allocation and will blindly draw a microchip from the opaque bag. The microchip will be implanted subcutaneously in the selected puppy. Once the microchip is implanted, the table on the Allocation Visit form will be consulted for the microchip number and allocation group letter. A syringe with the corresponding allocation group letter will be selected from the cool box and the contents injected into the puppy.

This method will conceal the sequence until interventions are assigned to *n*-1 puppies. The allocation sequence will not be concealed for the last or only puppy in a litter, but this will not create selection bias as long as all *n* puppies present are enrolled. This method will maintain owner blinding, as owners will not be aware of the allocation group letter meanings nor the contents of the syringes. Study personnel will not be blind to intervention allocation.

#### Implementation

Study field team leaders will generate the allocation sequence and prepare the study interventions, microchips and Allocation Visit form. Another member of the study field team will assign puppies to interventions. The study field team leader will hand the completed Allocation Visit forms to the Study Manager, who is not part of the field team, who will enter the allocation data in a password-protected spreadsheet. The completed allocation table will be removed from the Allocation Visit form by the Study Manager and stored in a locked filing cabinet in an area with restricted access (Study Manager’s office), separate from the Weekly Study visit forms. This will maintain analytical blinding until final data analysis, when the allocation data will be merged with the outcome data, using the microchip number as the unique identifier.

#### Who will be blinded

Owners will be blinded to assignment to interventions until exit of their puppies from the study. Data analysts will be blinded after assignment to interventions by maintaining outcome data in a separate database to allocation data. These datasets will be locked and merged after exit from the study of the last study subject, for final data analysis. Although the study field personnel will not be blinded to intervention allocation, they will not have access to this information after the allocation data is removed from the Allocation Visit form.

#### Procedure for unblinding if needed

Unblinding of owners will occur if the puppy is exposed to a suspect rabid animal, or if the puppy itself is a suspect rabies case. In these cases, knowledge of vaccination status is important for the management of the rabies risk. Suspect rabies cases will be defined as the observation or report of abnormal behaviour or suspected clinical signs in a live dog (including but not limited to salivation, central nervous system signs, ataxia, paresis/paralysis, convulsions, and/or coma). Information on the case, including vaccination status, will be provided to the office of the Sub-County Veterinary Officer as soon as possible after detection. Puppies whose owners are unblinded to their intervention allocation will be removed from the study (censored) at the time of unblinding.

### Interventions

#### Details of investigational products

##### Adjuvanted rabies vaccine (ARV)

Defensor® 3, Zoetis (adjuvanted rabies vaccine licensed for use in dogs, cats, cattle and sheep in Kenya; registration number VMD 2018/200078–11). The vaccine is packaged in liquid form and presented in 10-dose vials. The vaccine potency is ≥ 1.0 IU/mL (measured according to the international standard and the National Institutes of Health test). The dose is 1 mL (as directed on the package insert). The route of administration will be via subcutaneous injection. Storage is between + 2 °C and + 7 °C.

##### Non-adjuvanted rabies vaccine (NRV)

Rabies TC vaccine, KM Biologics Co., Ltd., 6 1 Okubo 1, Kita ku, Kumamoto City, 860 8568 Japan (non-adjuvanted inactivated tissue culture rabies vaccine licensed for use in dogs and cats in Japan; registration number 26 No. 3430). The vaccine is packaged in liquid form and presented in 10-dose vials. Each 10 mL vial contains not less than 10^8.5^ TCID_50_ RC-HL rabies virus strain, inactivated with beta-propiolactone and purified by macrogol treatment, and not more than 1.0 mg thimerosal as preservative. The dose is 1 mL (as directed on the package insert). The route of administration will be via subcutaneous injection. Storage is between + 2 °C and + 10 °C, away from direct sunlight.

##### Placebo

Sterile saline for injection (sodium chloride 0.9%, Sabax), packaged in liquid form and presented in 5 mL ampoules. The dose is 1 mL. The route of administration will be via subcutaneous injection. Storage is at room temperature. Injection of a placebo substance in dogs allocated to the control group will assist to mask the intervention from the owner. Subcutaneous injection of 1 mL of normal saline is not anticipated to affect the outcome or potential mediating variables in this study.

#### Intervention description

Eligible puppies will be randomized in a 1:1:1 ratio between ARV, NRV or placebo, receiving a single subcutaneous injection between the scapulae of 1 mL, using a 2–3 mL syringe and 21-23G needle. The injection will be done after the blood draw, on Study Day 0.

After opening, the vaccines will be used immediately or within 72 h if kept at between 2 °C and 8 °C. Opened vaccines (ARV and NRV) will be disposed of after 72 h or within 60 min if kept at room temperature. Unused reconstituted vaccines or expired vaccines will be treated as biological waste and disposed of through the Kenya Medical Research Institute (KEMRI) laboratory biomedical waste system. Unused saline will be discarded after first use of the ampoule, through the non-biological waste system.

### Relevant concomitant care permitted or prohibited during the trial

Use of concomitant veterinary treatments by owners will not be restricted. Owner-reported use of concomitant veterinary treatments will be captured during biweekly visits in weeks 1, 3, 5, and 7 post-intervention (p.i.).

### Adverse event reporting and harms

Occurrence of adverse events following intervention will be assessed by observation of subjects for 45 min after injection, physical examination of procedure sites by study field team leaders (sites of injection, microchip implantation and venipuncture) and soliciting reports of adverse events from owners at follow-up visits at 24 h, 7 days, 21 days, 35 days and 49 days p.i.. Based on risk information previously described for the study interventions, the occurrence of the following reactions will be assessed during post-intervention observation, physical examination and solicitation of owner reports:

- Local reactions (limited to the site of the injection): pain, erythema, oedema, pruritus and induration.
- Systemic reactions: fever, lethargy, inappetence.
- Hypersensitivity or allergic reactions: urticaria (‘hives’), erythema and/or angioedema (swelling) of the face and muzzle; hypersalivation; generalized pruritus; vomiting; diarrhea; dyspnea (difficulty breathing); wheezing; coughing; collapse; death.

Reactions will be considered adverse events if they meet any of the following criteria:

- Swelling at the site of injection larger than 2 cm in diameter at any time point.
- Swelling at the site of injection that increases in size by more than 50% between visits.
- Swelling at the site of injection that is still present at 35 days p.i., irrespective of size.
- Injection site that is painful and warm to the touch at any time point.
- Any combination of signs of local and systemic reactions at any time point beyond 24 h p.i.
- Any signs of hypersensitivity or allergic reactions occurring within 24 h of administration of study intervention.

Adverse events will be reported to the Study Monitor, the RUSVM Attending Veterinarian and JOOUST Ethics Review Committee (ERC) by the principal investigator within 48 h of observation of the event or (in the case of owner-reported events) receipt of a report of an event by the study team. At the end of the study, any adverse events established will be reported to RUSVM Institutional Animal Care and Use Committee (IACUC). Vaccine-associated adverse events will also be reported to the registration holders.

### Animal management and housing

Animals will remain with their owners for the duration of the study, and upon exit from the study.

### Schedule of events

**Fig. 2:**
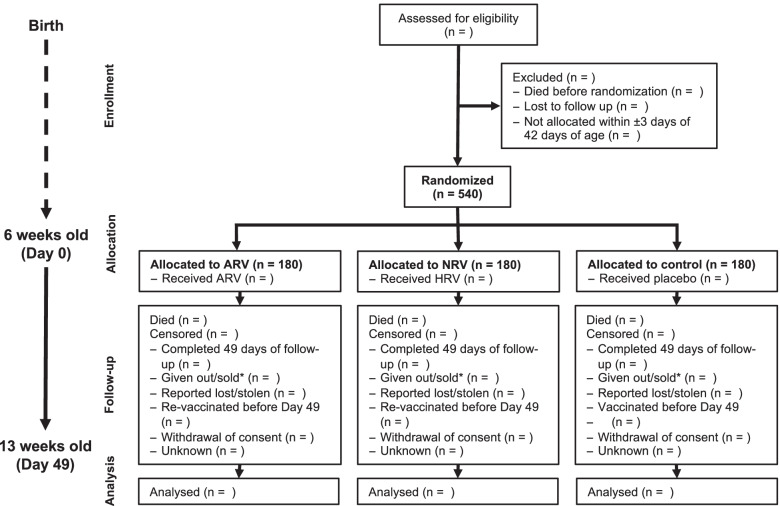
Flow diagram of study subjects. The numbers for ‘*n* = ’ will be determined in the study. *Given out or sold outside of the study site (33 villages), or new owner does not provide consent

Figure 2 shows the flow diagram of study subjects. Table 1 shows the schedule of key events. Eligibility of adult female dogs will be established and owners enrolled before the birth of puppies. To accurately capture births of puppies, adult females will be followed up monthly until oestrus or pregnancy, fortnightly from oestrus until pregnancy, and weekly from pregnancy. Litters will be registered at birth (birth date, number and sex of puppies) during a household visit. Litters of puppies will be followed up through weekly household visits from birth to 42 days of age, and deaths and losses to follow-up recorded. A few days before 42 days of age, the owner will be contacted and a household visit scheduled for collection of baseline data, allocation and intervention in puppies at 42 days of age, with a 7-day window (from 3 days before to 3 days after the due date). On study day 0 (week 0), puppies will be implanted with a subcutaneous 8 mm × 1.4 mm radio frequency identification microchip for unique and permanent identification, randomly allocated to a treatment group, and administered the allocated treatment by subcutaneous injection. After the allocation and intervention visit, a follow-up visit and blood collection will be done 24 h later, with a 7-h window (from 3 h before to 3 h after the appointed hour). Thereafter, weekly visits will continue to capture deaths and losses to follow-up (weeks 1–7 p.i.). Additional data collection will be done at the weekly visits in weeks 1 (7 days p.i.), 3 (21 days p.i.), 5 (35 days p.i.) and 7 (49 days p.i.), with a 5-day window for the first visit (from 2 days before to 2 days after the due date) and a 7-day window for visits thereafter (from 3 days before to 3 days after the due date). The study exit visit will be in week 7 (49 days p.i.), with a 7-day window (from 3 days before to 3 days after the due date). Sample collection (blood and rectal swabs) will be done at baseline, 24 h p.i. (blood only), and in weeks 1, 3 and 5.

**Table 1:** Schedule of key events

	Pre-birth	Birth cohort follow-up						NERVE-Dog RCT								
Age (weeks)		0	1	2	3	4	5	6		7	8	9	10	11	12	13
Age (days)		0	7	14	21	28	35	42	43	49	56	63	70	77	84	91
Study week		-6	-5	-4	-3	-2	-1	0		1	2	3	4	5	6	7
Days from intervention		-42	-35	-28	-21	-14	-7	0	1	7	14	21	28	35	42	49
**Owner informed consent**	**X**															
**Adult female rabies vaccination**	**X**															
**Adult female follow-up**																
**Monthly until oestrus or pregnancy**	**X**															
**Fortnightly from oestrus**	**X**															
**Weekly from pregnancy**	**X**															
**Household visit**		**X**	**X**	**X**	**X**	**X**	**X**	**X**	**X**	**X**	**X**	**X**	**X**	**X**	**X**	**X**
**Birth registration**		**X**														
**Microchip implant**								**X**								
**Allocation**								**X**								
**Intervention**								**X**								
**Assessment**																
Death		X	X	X	X	X	X	X	X	X	X	X	X	X	X	X
Loss to follow-up		X	X	X	X	X	X	X	X	X	X	X	X	X	X	X
Management and care^a^								X								
Weight								X		X		X		X		
Abdominal girth								X		X		X		X		
Aptitude test								X		X		X		X		
Concomitant care								X		X		X		X		X
Adverse events									X	X	X	X	X	X	X	X
**Blood collection**								**X**	**X**	**X**		**X**		**X**		
Cytokine assays								X	X							
Haematology								X				X				
Haemoparasite qPCR								X				X				
Anti-rabies IgG isotyping^b^												X				
**Rectal swabs**								**X**		**X**		**X**		**X**		
Viral qPCR								X				X				
Parasite qPCR								X				X				
**Study exit**																**X**

^a^Management and care assessment will be repeated if the subject changes ownership during follow-up

^b^ARV and NRV arms only

### Assessment of outcomes

Assessment of the primary outcome (survival time) will be done during weekly visits to the households of study dogs. Visits will be supplemented by unscheduled contact with owners outside of weekly visits (phone calls to/from owners; chance meetings with owners). Measurement of death will rely on owner reports, verified by study personnel. Deaths will be classified as confirmed or unconfirmed. Deaths verified by study personnel through observation of the carcass and confirmation of the identity of the puppy will be recorded as confirmed deaths. Owner-reported deaths that are not verified by study personnel will be recorded as unconfirmed deaths. The date of death will be determined by owner interview.

### Pathological outcomes

A full necropsy to establish the cause of death will be performed in all cases of death post-intervention in which the study team is notified of the death by the owner and can retrieve the carcass before post-mortem decomposition is too advanced for necropsy. A post-mortem examination will be carried out at the project field office in central Asembo (Lwak village) for any deaths that occur post-intervention. Only personnel with current vaccination against rabies will perform post-mortem examinations. Appropriate personal protective equipment will be worn by personnel conducting the post-mortem examination. For suspect rabies cases, this will include a face shield and gloves. The Sub-County Veterinary Office will be notified in these cases and a post-mortem examination will only be performed with the Sub-County Veterinary Officer’s permission. In all cases, the brain will be removed from carcasses and halved longitudinally. Half of the brain will be placed in glycerol-saline and half in 10% buffered formalin. Other tissue specimens will also be collected and placed in 10% buffered formalin. Storage until transport will be at the Project field office (Lwak) in the research storeroom with access control, within a secondary plastic container, to prevent spillage. Specimens in 10% buffered formalin will be transported to JOOUST for histopathological examination.

Additional tissue specimens including but not necessarily limited to the small intestine, spleen, lung and brain will be collected during the post-mortem examination and placed in RNAlater™. Nucleic acid will be purified from these specimens at the KEMRI Kisian campus. Purified nucleic acid will be analysed at KEMRI by quantitative PCR (qPCR) for viral (CPV/CDV) and protozoal/bacterial (*Babesia/Ehrlichia/Anaplasma*) pathogens. Purified nucleic acid may might also be transported to the University of Pretoria for further molecular analyses.

Brain samples in glycerol-saline of all enrolled puppies that die during the project between 42 and 91 days of age and from which specimens can be obtained will be sent to the Central Veterinary Laboratories (CVL, Kabete) for rabies diagnostic testing. All brain specimens for rabies diagnostics will be submitted through the Siaya County Director of Veterinary Services (CDVS’s) office. All rabies test results will be communicated by the CVL Rabies Laboratory directly back to the CDVS and from there forwarded to the research team.

### Laboratory assays

Sample collection (blood and rectal swabs) will be done at baseline (study day 0), 24 h p.i. (blood only), and at the weekly visits in weeks 1 (7 days p.i.), 3 (21 days p.i.) and 5 (35 days p.i.).

For blood samples, puppies’ circulating blood volume (CBV) will be conservatively estimated from their weight as 50 mL/kg (normal circulating blood volume for a dog is 85 mL/kg). We will collect a maximum of 4% of the CBV in a single draw, and a maximum of 9% in one week, followed by two weeks’ rest. For a puppy of average weight at 6 weeks (2 kg), this amounts to a maximum of 4 mL (4% of 50 mL × 2 kg) in a single draw and 9 mL in one week. Collection of blood will be done with a 2–5 mL syringe and a 20–23 gauge needle from the jugular or cephalic vein and transferred into EDTA and serum-separator tubes. Table 2 shows the schedule of blood draws.

**Table 2:** Schedule of blood draws

	**Day 0 (baseline)**	**Day 1 (24 h p.i.)**	**7 days p.i**	**21 days p.i**	**35 days p.i**
**Total Volume**^a^	**3–4 mL**	**1 mL**	**3–4 mL**	**4–5 mL**	**4–5 mL**
**EDTA tube volume**	2.5–3.5 mL	1 mL	2.5–3.5 mL	2.5–3.5 mL	2.5–3.5 mL
Thin blood film	X	-	X	X	X
Plasma extraction (1.5 + mL)	X	X	X	X	X
Haematology aliquot^b^ (0.5 mL)	X	-	-	X	-
qPCR aliquot (0.5 mL)	X	-	-	X	-
Storage aliquot (0.5 mL)	X	-	X	X	X
**Serum tube volume**	0.5 mL	-	0.5 mL	1.5 mL	1.5 mL
Anti-rabies IgG isotyping^c^ (1 mL)	-	-	-	X	-
Storage aliquot (0.5 + mL)	X	-	X	X	X

^a^Determined by the weight of the dog taken before the blood draw. Values shown in the table are ranges from a preliminary study

^b^500 μL of sample from an IDEXX VetCollect tube or 650 μL from a 1.3 mL Sarstedt tube (analyzer aspirates 30 μL of sample)

^c^Subset of subjects inARV (*n* = 9) and NRV (*n* = 9) arms only

Blood samples will be placed in an ice bath and transported to the laboratory at KEMRI. On days 0, 7, 21 & 35 p.i., a thin blood film will be prepared. On samples from days 0 and 21 p.i. an aliquot of blood in EDTA will be used for haematology analysis. A second aliquot will be stored in RNAlater™ for later nucleic acid purification and qPCR. A third aliquot of blood in EDTA will be stored at -80 °C. The remaining blood (and all blood at 24 h p.i.) in EDTA will be centrifuged and plasma extracted. Blood in serum-separator tubes will be centrifuged and serum extracted and stored in aliquots for IgG isotyping (day 21 p.i.) and storage at -80 °C (days 0, 7, 21 & 35 p.i.).

For plasma cytokine assays, we will conduct an internal pilot study on plasma samples on days 0 and 1 from the first 9 subjects in each vaccine:sex group (36 subjects/72 samples in total), using the Merck CCYTMG-90 K-PX13 Milliplex map canine cytokine to identify changes in Th1/Th2-directing plasma cytokines concentrations. Information from this pilot study will inform the choice of four cytokines that will be measured in all study subjects (days 0 and 1) using the R&D Systems Canine DuoSet ELISA.

We will confirm the Th1/Th2 balance of the rabies-specific immune response in a subset of subjects in the ARV (*n* = 9) and NRV (*n* = 9) treatment groups by measurement of IgG antibody isotypes (IgG1 vs IgG2a) 21 days p.i..

Rectal swabs will be taken at baseline, 7, 21 and 35 days p.i. Swabs will be placed in RNAlater™ and stored at -80 °C until further processing.

Nucleic acid will be purified from EDTA blood in RNAlater™ and from rectal swabs in RNAlater™ at KEMRI. Extracted DNA/RNA will be analysed at KEMRI by quantitative PCR. Purified nucleic acid may also be transported to the University of Pretoria or to RUSVM for further molecular analyses.

### Statistical analysis

#### Primary objective

The effect of the treatment allocation on survival time will be estimated using a mixed-effect Cox proportional hazards model with litter as a random effect, and including sex as a covariate, for (i) ARV compared to placebo and (ii) NRV compared to placebo. To evaluate a differential effect of treatment between males and females, we will include an interaction term between sex and treatment group. The statistical significance of the interaction term will be evaluated using the likelihood ratio test.

#### Sample size estimate

The study is powered to detect a hazard ratio for the interaction effect of treatment by sex, equivalent to that observed in a previous study (0.28) [43]. The proportion of puppies dying during the follow-up period is also estimated from the previous study (0.22). The proportion of puppies in each treatment:sex category is anticipated to be equal, based on an equal allocation ratio and the observation in the preliminary study of equal sex ratio at 42 days of age. Assuming a significance level of 0.05, 80% power, and an expected loss to follow up of 25%, the estimated sample size required to detect the postulated hazard ratio is 180 per treatment group (540 in total). No adjustment for multiplicity is applied.

To enroll 540 puppies at 42 days of age, we anticipate registering approximately 1,080 births (to account for the approximately 50% loss to follow up between birth and 6 weeks of age, based on our previous study, [43]). With an average litter size at birth of 6.19 in the previous study, this represents 175 litters. In the previous study, we registered 114 litters at birth from a cohort of 151 adult female dogs. Thus, to register 175 litters (1,080 puppies at birth), we anticipate enrolling a cohort of 232 adult females in 186 households.

#### Secondary objectives

For the Th1/Th2 balance score (secondary objective 1), we will follow the method of Genser et al. [49] to aggregate the cytokine measurements into a summary score of the immunological response (Th1/Th2 balance score). Briefly, we will group the variables into Th1-priming (e.g. IFN-γ, TNF-α) and Th2-priming (e.g. IL-4, IL-10) and perform exploratory data analysis of individual measurements. We will visually explore bivariate associations within groups and determine an appropriate method for interdependence analysis and data aggregation to produce a summary measure of Th1 and Th2 responses. Finally, we will group the data into a Th1/Th2 balance score with higher values referring to Th2 skewness. We will confirm the Th1/Th2 balance of the rabies-specific immune response in the ARV and NRV treatment groups by measurement of IgG antibody isotypes (IgG1 vs IgG2a) 21 days p.i.

For the mediation analysis in the RCT (secondary objectives 1, 2 and 3a to 3c), we will use the method of [50] and [51] for mediation analysis for multiple mediators with time-to-event data to estimate (i) the proportion (with 95% CIs) of the effect of ARV compared to placebo on survival time that is mediated through each of the potential mediators (Th1/Th2 balance score, plasma cytokine concentrations, body weight, abdominal girth, aptitude test score), in males and females separately, and (ii) the proportion (with 95% CIs) of the effect of NRV compared to placebo on survival time that is mediated through each of the potential mediators (plasma cytokine concentrations, body weight, abdominal girth, aptitude test score), in males and females separately.

For secondary objectives 3d to 3 h, we will conduct a causal mediation analysis of an NCC study within the RCT. We define a case as a death between 21 and 35 days post-injection This period was chosen because the majority of deaths in vaccinated females occurred 21–35 days post-injection in a previous study [43]. Controls will be randomly selected from among subjects still at risk at case event times, with a sampling ratio of cases:controls of 1:2. Samples collected at baseline and at 21 days post-injection would be analysed for these subjects for the following mediator values: (i) quantitative PCR of *Babesia* spp., *Ehrlichia canis* and *Anaplasma platys* from blood and of CPV, CDV, *Ancylostoma caninum* and *Toxocara canis* from rectal swabs [52–57]; (ii) 27 whole-blood parameters including haematocrit, erythrocyte count, haemoglobin concentration, erythrocyte mean cell volume, reticulocyte count, and reticulocyte haemoglobin content measured by haematology analyser; and (iii) levels of CRP in plasma [58]. For this study design, samples must still be collected and stored from all subjects at the timepoints of interest (days 0 and 21 p.i.), but only samples from subsequent cases and controls will be analysed. Due to its time-sensitive nature, haematology will be done on all subjects on days 0 and 21 p.i.. For the causal mediation analysis of the NCC, we will use the method described in Kim et al. [59].

For secondary objective 4, causes of mortality will be assigned based on post-mortem examinations of carcasses and owner interviews (verbal autopsy). Cause-specific mortality rates will be calculated as the number of deaths assigned to a specific cause per 1,000 dog-years at risk. Cause-specific mortality rate ratios with 95% confidence intervals (CIs) will be used to compare between treatment groups and by sex.

For secondary objective 5, the effect of sex on survival from birth to six weeks of age will be presented as the estimated hazard ratio of females compared to males from a mixed-effects Cox proportional hazards model with litter as a random effect, along with 95% CIs.

## Discussion

The status quo as it pertains to the theory of detrimental NSEs of non-live vaccines is one of polarized viewpoints [18], with debate between protagonists and antagonists largely focused on the robustness and risk of bias of existing data, which will only be resolved by randomized trials of relevant outcomes in low-income settings. We are not aware of any such trials planned to examine potential deleterious NSEs of non-live vaccines such as DTP, presumably due in large part to ethical challenges of random allocation of children to a control group—an issue likely exacerbated by the lack of consensus regarding a state of clinical equipoise, thereby perpetuating the status quo. Furthermore, there are no published studies that demonstrate a mechanistic link between immunological changes following vaccination and clinically-relevant NSE outcomes in populations [18]. This protocol focuses on the establishment of a valid animal model for randomized controlled studies at low risk of bias in high-mortality populations, which will allow for the integration of studies of mechanistic links between vaccine exposures, immunological changes and survival outcomes. This will open new horizons for causal inference in the study of NSEs of vaccines, through which scientific consensus on the topic can be achieved.

Identifying mechanisms of action of any deleterious non-specific effects of vaccines will be essential to mitigate these effects. Causal mediation analysis is a technique to quantify possible mechanisms (causal pathways) linking an exposure to an outcome, by decomposing the total effect of the exposure on the outcome into a natural indirect effect (NIE; acting through the measured mediator/s) and a natural direct effect (NDE; acting through all pathways other than the measured mediator/s) [60]. An NCC study design reduces costs and effort involved in determining mediator values for all subjects at each time point in the RCT and allows more efficient estimation of NIEs and NDEs by using mediator values only for selected cases and controls [59]. For reasons of cost and efficiency, in this study we will focus the NCC on deaths between 21 and 35 days p.i, with measured mediators at time points at days 0 and 21 p.i. Specimens collected at other time points will be stored for possible future analysis in a broader causal mediation analysis study.

Based on our hypotheses, we expect that, among the placebo group, males will have higher mortality, higher pathogen loads and more severe disease compared to females. Among females, we expect that there will be no difference in mortality between the NRV and placebo groups, but that the ARV group will have higher mortality, again mediated by higher pathogen loads and more severe disease. We anticipate that these changes are preceded by shifts in key serum cytokine concentrations towards an anti-inflammatory immune response in females. If confirmed, these results will provide a rational basis for mitigation of detrimental NSEs of non-live vaccines in high-mortality populations.

## Data Availability

Not applicable.

## Abbreviations

ARV: Alum-adjuvanted rabies vaccine
BCG: Bacillus Calmette-Guérin
CBV: Circulating blood volume
CDV: Canine distemper virus
CDVS: County Director of Veterinary Services
CPV: Canine parvovirus
CRP: C-reactive protein
CVL: Central Veterinary Laboratories
DTP: Diphtheria-tetanus-pertussis
EDTA: Etheylenediaminetetraacetic acid
ELISA: Enzyme-linked immunosorbent assay
ERC: Ethics Review Committee
HDSS: Health and Demographic Surveillance System
IACUC: Institutional Animal Care and Use Committee
JOOUST: Jaramogi Oginga Odinga University of Science and Technology
KEMRI: Kenya Medical Research Institute
MCV: Measles-containing vaccine
NCC: Nested case–control
NDE: Natural direct effect
NIE: Natural indirect effect
NRV: Non-adjuvanted rabies vaccine
NSE: Non-specific effect
OIE: World Organisation for Animal Health
p.i.: Post-intervention
qPCR: Quantitative polymerase chain reaction
RCT: Randomized controlled animal trial
RUSVM: Ross University School of Veterinary Medicine
WHO: World Health Organisation
